# Supplementary material for: Curated and harmonised transcriptomics datasets of interstitial lung diseases
Source: Data Brief. 2025 Oct 14;63:112139. doi: 10.1016/j.dib.2025.112139 (PMC12581653; doi:10.1016/j.dib.2025.112139)
Supplement: Supplementary file 1 [file mmc1.zip › Supplementary_material/DNA-microarray/GSE53845/GSE53845_eUTOPIA_Agilent_QC_Report_2023-12-15.tar.gz!/Users/OWNER/AppData/Local/Temp/RtmpAFKb7F/2023-12-15/ma.pdf]

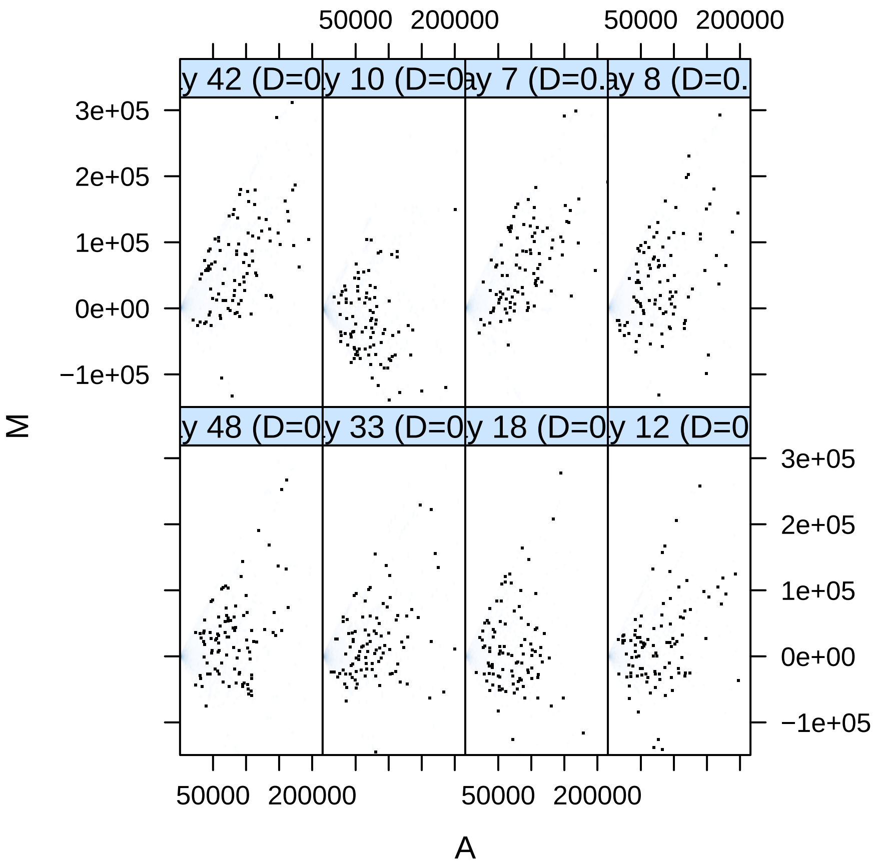

y 42 (D=0
y 10 (D=0
ay 7 (D=0.
ay 8 (D=0.
y 48 (D=0
y 33 (D=0
y 18 (D=0
y 12 (D=0
50000
200000
3e+05
2e+05
1e+05
0e+00
−1e+05
M
A
